# Appraising Artificial Intelligence Studies in Dental and Maxillofacial Imaging: A Task-Routed Framework for Reporting Completeness and Risk of Bias

**DOI:** 10.7759/cureus.117978

**Published:** 2026-10-15

**Authors:** Volodymyr Kachmar, Roksolana Mikhlin, Tamara Danko, Alina Shmal

**Affiliations:** 1 Dentistry, Tufts University School of Dental Medicine, Boston, USA; 2 Dentistry, Danylo Halytsky Lviv National Medical University, Lviv, UKR; 3 Dentistry, Tufts University, Boston, USA; 4 Dentistry, JuALDent s.r.o., Stary Materov, CZE

**Keywords:** artificial intelligence in dentistry, cone-beam computed tomography (cbct), deep learning algorithms, dental digital radiography, diagnostic accuracy of ai, meta-research, reporting guideline, risk of bias

## Abstract

Artificial intelligence studies in dental and maxillofacial imaging use different designs and endpoints, but reviews often apply a single adapted appraisal instrument across them. Recent updates to reporting guidelines and risk-of-bias tools have clarified their respective scopes. This narrative methodological review proposes an appraisal framework in which the review question, study design, and each eligible evaluation determine the instrument used. Diagnostic accuracy estimates are assessed using appropriate criteria, with comparative accuracy evaluations assessed separately according to relevant methodological guidance. Diagnostic or prognostic multivariable models are evaluated for risk of bias and methodological quality, with model development quality and evaluation risk of bias reported separately. Structural segmentation, localization, registration, and image-enhancement evaluations receive a declared descriptive appraisal where the framework provides no established task-specific risk-of-bias route. Reporting completeness is assessed separately using the relevant reporting guidelines. Hybrid reports may contribute to more than one route, with evaluations and estimates linked to the underlying study and dataset. Results are presented by route, with explicit denominators, rather than combined into a single quality score. The proposed extraction items address annotation provenance, patient-level separation between data partitions, within-patient clustering, dataset reuse, clinical exclusions, and modality-specific acquisition characteristics. The review also identifies limitations concerning general-purpose multimodal models and certainty-of-evidence assessment. The framework offers practical guidance and a proposed minimum reporting set for authors, reviewers, and editors. Its routing reliability has not been evaluated; therefore, the framework is intended to improve transparency and comparability and requires empirical testing.

## Introduction and background

Dental reviews describe a recurring development-and-testing design: a convolutional network is trained on a retrospective image collection, often from one center, and evaluated on a held-out subset [1,2]. Such studies have investigated caries detection, periodontal bone loss assessment, periapical lesion identification, tooth detection and numbering, cephalometric landmarking, third molar assessment, and implant planning. This pattern remains relevant to appraisal, although it does not describe every current study, particularly those evaluating general-purpose multimodal models without task-specific training. 

Design determines the clinical stakes. A caries classifier that performs well only on the scanner, patient population, and annotation convention represented in its development data may miss lesions or prompt unnecessary restorative intervention when used elsewhere. Published evidence informs clinical and regulatory decisions, but incomplete reporting of partitioning, reference standards, and test-set provenance limits how thoroughly that evidence can be assessed.

Medicine encountered this problem earlier and has documented it thoroughly. In a review of 82 studies comparing deep learning with healthcare professionals on medical images, 25 reported external validation, and only 14 of those compared the model and clinicians on the same external sample [3]. A systematic review of design, reporting, and claims in deep learning studies identified two published randomized trials and 81 non-randomized comparisons, most at high risk of bias, with abstracts that made claims exceeding their evidence [4]. When the reviews themselves were audited, more than half of the primary studies pooled across artificial intelligence diagnostic accuracy systematic reviews carried a high or unclear risk of bias in patient selection, and QUADAS-2 (Quality Assessment of Diagnostic Accuracy Studies, Version 2) had been applied inconsistently across reviews [5]. In a review restricted to externally validated radiologic deep learning algorithms, 70 of 86 algorithms showed some decrease in performance on external data [6]. Dentistry has its own direct evidence: a model trained on radiographs from one country lost substantial performance when applied to data from another [7].

Appraisal rests on two questions that are often conflated. Reporting completeness asks what an author wrote: whether the paper states enough for a reader to understand what was done. Risk of bias asks what the study design permits us to conclude: whether features of the design, conduct, or analysis could have distorted the result. A third question, applicability, asks whether the participants, index test, and reference standard match the clinical question a reader has in mind. A well-conducted study can be poorly reported, and a thoroughly reported study can be badly designed. Reporting checklists answer the first question; risk-of-bias tools answer the second and third questions. Keeping them apart is the organizing principle of this review.

Medicine's response has been an unusually complete set of instruments for both, and four of them changed within two years: the Checklist for Artificial Intelligence in Medical Imaging (CLAIM) was revised in 2024 [8,9]; the Prediction model Risk Of Bias ASsessment Tool gained an artificial intelligence extension (PROBAST+AI) in 2025 [10]; the Standards for Reporting of Diagnostic Accuracy studies gained one, STARD-AI, in the same year [11]; and the QUADAS tool moved from its second to its third version in 2026 [12,13]. What each change involves is set out in this review.

Dental appraisal practice illustrates both instrument adaptation and variation in published judgements. A review of deep learning for caries detection found that 11 of 42 studies showed low risk of bias across all QUADAS-2 domains [14]. A 2025 review of caries detection on intraoral images, using a modified QUADAS tool, rated 19 of 23 studies at low risk and four at high risk [15]. A review of dental plaque and gingivitis on oral photographs found low risk across all four QUADAS-2 domains in 11 of 23 studies and low applicability concern across all domains in nine [16]. A 2026 review of Faster R-CNN and Mask R-CNN studies described its assessment as QUADAS-AI and summarized applicability concerns as generally low, while reporting high or unclear participant-selection applicability concern in 13 of 17 studies [17].

These findings concern partially overlapping literature with different tasks and eligibility criteria; they are not direct reassessments of the same corpus. Publication windows, populations, modalities, target conditions, reviewer interpretation, and instrument selection and adaptation may all contribute to the differences. Without independently reappraising the same primary studies, their relative contributions cannot be isolated. Instrument heterogeneity is a plausible contributor that an explicit routing procedure could help address.

Instrument scope is a central difficulty. Dental reviewers are being asked to apply a diagnostic accuracy tool to a literature in which many studies report no diagnostic accuracy estimates at all. A segmentation study reporting a Dice coefficient, a landmark study reporting mean radial error, and a prognostic model predicting implant failure are not diagnostic accuracy studies, and forcing them through QUADAS-2 produces a judgement of unclear meaning. QUADAS-3 makes this explicit by tying assessment to a selected accuracy estimate: where no such estimate exists, the tool does not apply.

The gap this review addresses is therefore specific: instrument selection and adaptation need to be made explicit when dental imaging artificial intelligence studies with different designs are appraised together. The illustrative reviews do not establish how often routing decisions are reported across the whole field. They do show why a review should explain which instrument applies to each eligible evaluation and how assessments made with different tools will be presented.

This review sets out an alternative. It describes what changed in the appraisal landscape and why it matters for dentistry; proposes that appraisal be routed by study task rather than applied uniformly; specifies the routes and their boundaries; identifies the reference standard, analysis unit, and acquisition characteristics that dental appraisal must capture and that general medical instruments do not name; and translates the framework into practical guidance for authors, reviewers, and editors. It is a methodological review, not a systematic review of primary studies. It reports no corpus-level estimates of adherence or bias, and the framework it proposes has not yet been tested for routing reliability.

## Review

How this framework was developed

This is a narrative methodological review, not a systematic review of primary studies. The initial identification of instruments and illustrative dental reviews was documented in the authors' search record dated 24 September 2026. Instruments were identified through the EQUATOR Network reporting-guidelines library, reference lists, and targeted bibliographic lookups. For this revision, publication details were checked against Crossref and PubMed, with instrument lookups updated on 30 September 2026. Appendix 1 provides the identification approach, queries, dates, and limits, distinguishes the historical search record from the revision checks, and documents the broad PubMed retrieval discussed under Scale. Illustrative reviews were selected to show different tasks, modalities, and appraisal practices; no exhaustive sample or corpus-level synthesis is claimed. The routing rules were developed by comparing the stated scopes of the instruments with the designs and endpoints represented in these examples. Where no established route was identified within this framework, a descriptive appraisal was specified instead of assigning a risk-of-bias label. Three hypothetical worked examples explain how the rules apply. During revision, all four authors independently reviewed and confirmed the AI-assisted editorial assessments. This author verification is distinct from a formally documented pre-consensus human routing-reliability study; no such dataset or formal consensus study is reported.

The appraisal landscape changed four times in two years

CLAIM was introduced in 2020 as a 42-item guide for authors and reviewers of medical imaging artificial intelligence studies, modelled on the STARD framework [8]. It was adopted widely and, in the process, was frequently repurposed as a scoring instrument, a use its authors did not intend. The 2024 update expanded the checklist to 44 items with official response options of Yes, No, and Not Applicable, and explicitly addressed the drift toward score-like use [9]. An analysis of studies citing CLAIM found that self-reported adherence was often declared without a completed checklist being supplied [18]. Reviewers scoring a corpus that spans the revision must decide which version applies to which paper. We would key that decision to the date a manuscript was submitted, where journals report it, rather than to the date it appeared: a paper submitted before the 2024 update could not have used it, and a rule based on publication date penalizes authors for a review and production interval they did not control. Where submission dates are unavailable, publication date is the only workable fallback and the choice should be stated, since it will misclassify some papers. Both facts matter for dentistry: adherence should be reported as the proportion of applicable items reported, without thresholds separating "good" from "poor" studies, and a declaration of adherence in a manuscript is not evidence that the checklist was completed.

Dentistry has had its own reporting instrument since 2021. A 31-item checklist for artificial intelligence in dental research was developed by the International Association for Dental Research e-oral health network together with the International Telecommunication Union and World Health Organization focus group on artificial intelligence for health, and is listed by the EQUATOR Network library of reporting guidelines (https://www.equator-network.org/reporting-guidelines) [19]. It covers items that general imaging checklists treat generically, but that carry particular weight in dentistry, including the source and calibration of annotators and the clinical definition of the target condition. It complements rather than replaces CLAIM, and the two can be assessed item by item at report level, with any reporting proportions presented separately.

PROBAST was introduced in 2019 for prediction model studies [10]. It could be applied to machine learning models, but its update responds to subsequent methodological developments, and reviews using it found that the overwhelming majority of machine learning prediction studies carried a high risk of bias, concentrated in the analysis domain [20]. PROBAST+AI, published in 2025, addresses this directly: it separates the appraisal of model development from the appraisal of model evaluation, addresses issues including data leakage and fairness, and retains a distinction between risk of bias and applicability [10]. When the appraisal concerns the development or evaluation of a dental prediction model for caries incidence, implant survival, or orthodontic treatment need, PROBAST+AI is the relevant route, and its reporting is governed by TRIPOD+AI rather than by an imaging checklist alone [21].

QUADAS-3 is the most consequential change for dental reviews because QUADAS-2, published in 2011 with its four domains of patient selection, index test, reference standard, and flow and timing, has been the default instrument in this field for a decade [22]. Four changes matter. Assessment moves from the study to an accuracy estimate, so a study reporting several target conditions, model versions, thresholds, or test sets requires the reviewer to state which estimate is being appraised. Its four risk-of-bias domains are Participants, Index Test, Target Condition, and Analysis; the last replaces the former Flow and Timing domain. The concept of an ideal test accuracy trial is introduced as the comparator against which real designs are judged. Overall judgements of risk of bias and applicability are formalized, and reviewers are required to record the rationale for each judgement [12,13].

Estimate-level assessment does not mean one estimate per study. Separate assessments are needed when multiple target conditions, test settings, or estimates are relevant to the synthesis questions [12,13]. For a route-level descriptive summary, this framework permits a prespecified rule to select one estimate per evaluation and synthesis question. That is an optional summary procedure, not a requirement of QUADAS-3, and it does not replace assessments needed for the review's substantive questions. Relevance to the synthesis question is established first; only then are the study's stated primary endpoint and prespecified test set used to resolve competing eligible estimates.

QUADAS-C, published in 2021, was developed as an extension of QUADAS-2 for comparative accuracy studies [23]. It covers comparisons of two or more index tests, including model versus clinician, model versus model, and assisted versus unassisted reading. A comparative judgement requires assessment of the accuracy of each test and additional questions about the comparison, such as paired or randomized allocation to tests and blinding between test interpretations. The current QUADAS guidance recommends QUADAS-C alongside QUADAS-3, with adaptation as described in the QUADAS-3 explanation and elaboration document [13]. The base-tool version and any adaptation therefore need to be recorded. QUADAS-C assesses comparative risk of bias, not applicability, and it is not intended for indirect comparisons between separate single-test studies.

Reporting of diagnostic accuracy gained its own artificial intelligence extension in 2025. STARD-AI was developed through a literature review, a scoping survey, a Delphi process, and a consensus meeting involving more than 240 international stakeholders, and it addresses the items that STARD 2015 leaves unspecified for algorithmic index tests: data provenance and partitioning, model version and availability, the conditions under which the algorithm was run, and how its outputs were converted into a diagnostic decision [11]. For a dental study reporting sensitivity and specificity for caries on bitewings, STARD-AI and CLAIM are complementary rather than alternatives: the first governs how the accuracy study is reported, the second how the model and its development are described.

Two further instruments need to be distinguished from the reporting guidelines and risk-of-bias tools above. QUADAS-AI was announced in 2021, and its development protocol was published in 2024 [24,25]. No final tool statement was identified in the targeted PubMed lookup updated on 30 September 2026. The dental review cited above describes its assessment as QUADAS-AI but cites the 2021 development announcement [17]. Its judgements therefore require interpretation in light of the instrument version and adaptations actually used; the label alone does not establish use of a finalized, standardized instrument.

The same review reports a mean Radiomics Quality Score (RQS) of 74.4% for ten radiographic-image studies, corresponding to 268 of 360 possible points [17]. RQS was developed for radiomics workflows, including feature extraction, feature reduction, robustness, and validation [26]. End-to-end Faster R-CNN or Mask R-CNN detection does not, by itself, constitute an engineered radiomic-feature pipeline. Some RQS items address broadly relevant design issues, whereas others require a radiomics-specific interpretation. Without evidence that the relevant workflows and item rules were applicable, the aggregate percentage should not be treated as a general quality measure for these detection networks or compared directly with percentages from conventional radiomics studies.

APPRAISE-AI is a 24-item quantitative tool spanning six domains of methodological and reporting quality [27]. Its development study evaluated 28 sepsis-prediction studies. That validation does not establish suitability for every dental segmentation, landmarking, tooth-identification, or registration task. Because APPRAISE-AI combines reporting and methodological constructs, its total is not a risk-of-bias judgement in this framework. If it is used, its full item set and intended scope should be identified; omitted or adapted items and the reasons for adaptation should be reported, and an adapted score should not be presented as the validated original. A custom feature list should be labelled as a descriptive appraisal.

What lies outside this framework

Three boundaries should be stated before the routes are described:

The framework covers primary studies that develop or evaluate a model. Trial protocols and reports of clinical interventions have additional reporting requirements: SPIRIT-AI (Standard Protocol Items: Recommendations for Interventional Trials-Artificial Intelligence) and CONSORT-AI (Consolidated Standards of Reporting Trials-Artificial Intelligence), respectively [28,29]. Systematic reviews of diagnostic accuracy are reported using PRISMA-DTA (Preferred Reporting Items for Systematic Reviews and Meta-Analyses of Diagnostic Test Accuracy Studies) [30]. These guidelines concern different reporting objects and are not substitutes for primary-study appraisal.

The framework also stops short of assessing certainty of a body of evidence. GRADE (Grading of Recommendations Assessment, Development and Evaluation) distinguishes certainty assessment from individual-study appraisal and has guidance for diagnostic tests and strategies [31]. One of the illustrative dental reviews used GRADE alongside QUADAS-2 [16]. Descriptive information from the structural and enhancement routes may inform a certainty assessment, but these routes do not supply a standardized risk-of-bias judgement by themselves. A review using such evidence would need a justified method for assessing its methodological limitations and an appropriate certainty framework. This review provides neither a validated mapping to GRADE domains nor a complete certainty assessment; developing and evaluating that mapping remains further work.

General-purpose multimodal models require further qualification. In a zero-shot evaluation, there may be no task-specific training split to inspect, while pretrained data, model weights, or changes to a hosted service may be inaccessible to investigators. Relevant methods include the exact model identifier and access date, prompts, settings, repeat runs, output-to-decision rules, and the independence of the evaluation dataset. TRIPOD-LLM provides reporting guidance for studies using large language models [32]. Where a multimodal model is evaluated as a specified diagnostic index test against a clinical reference standard, the diagnostic accuracy route may still be applicable; absence of local training does not by itself make diagnostic appraisal impossible. However, this framework has not been evaluated for foundation-model studies, and it supplies no dedicated solution for undisclosed pretraining contamination or service drift. Reviewers should state which questions can be assessed, which remain uncertain, and where an existing instrument's scope is insufficient.

One instrument cannot appraise every dental imaging artificial intelligence study

Dental imaging artificial intelligence is not a single design class. It contains at least four:

Diagnostic classification and detection studies evaluate a clinical target condition against a reference standard, for example caries on bitewings or periapical lesions on panoramic radiographs. Routing depends on the clinical question and endpoint, not simply on whether a metric is called accuracy, sensitivity, or area under the curve.

Structural segmentation, localization, tooth-identification, and registration studies report overlap or distance metrics, such as Dice similarity coefficient, intersection over union, or mean radial error, against an annotated ground truth. Tooth segmentation on cone-beam computed tomography, cephalometric landmarking [33], tooth detection and numbering [34], and mandibular canal tracing belong here. Many report no diagnostic accuracy estimate at all.

Prediction model studies estimate the probability of a clinical state or future outcome using multivariable models, often combining imaging with clinical variables. Reviews in endodontics and implant dentistry describe varied diagnostic and prediction applications and weaknesses in study design or reporting [35,36], and clear identification of the model-development and evaluation components is needed for appraisal.

Image-enhancement and synthesis studies report image quality metrics or downstream task performance and may have no clinical reference standard. Routine task-specific preprocessing and augmentation are recorded within the downstream evaluation; a separate enhancement route requires enhancement or synthesis to be a distinct evaluated output.

These classes are not mutually exclusive, and the boundary between the first and third needs care. A diagnostic prediction model estimates the probability that a condition is present now, which is also what a diagnostic classifier does; the distinction is not the target but the object of appraisal. Where a study presents a model as an index test and reports its accuracy against a reference standard at a stated threshold, the diagnostic route applies. Where the appraisal question concerns how a multivariable prediction model was developed or how it performs as a model, the prediction route applies. Missing calibration reporting is a reporting or methodological limitation within that route; it does not make the prediction model ineligible for PROBAST+AI [10].

Three hypothetical worked examples make this concrete; they do not constitute an empirical routing exercise. A study reporting sensitivity and specificity for proximal caries on bitewings against expert annotation, at a fixed operating point, is routed to QUADAS-3 for that estimate. A study combining radiographic features with caries risk variables to estimate three-year caries incidence, reporting a c-statistic and a calibration plot, is routed to PROBAST+AI, with the development and evaluation parts rated separately. A study that segments carious lesions and separately evaluates lesion detection against a clinical reference standard may enter both routes: the segmentation output is appraised descriptively, and the lesion-level accuracy estimate is appraised with QUADAS-3.

QUADAS-2 and QUADAS-3 are diagnostic test accuracy instruments. Applied to a segmentation study, the index test domain becomes ambiguous, the reference standard domain collapses into annotation quality, and the resulting "low" or "high" risk of bias label attaches to nothing well defined. An overlap metric, such as a Dice coefficient of 0.93, is not by itself a clinical diagnostic accuracy estimate, and a study achieving it may have no demonstrated clinical utility whatever [37]. For lesion boxes or inflammation maps, record whether the evaluated errors concern a clinically defined condition decision, spatial delineation against annotations, or separately evaluated components of both. If the report and the synthesis question do not resolve that distinction, retain an indeterminate routing decision and explain it. A corresponding mismatch occurs when a prediction model is appraised only as an index test: diagnostic appraisal does not replace the questions specific to prediction-model development and evaluation, particularly in the analysis component [20].

Instrument misfit offers one plausible explanation for divergent judgements. Applying a tool outside its scope can require locally chosen adaptations, reducing the comparability of assessments. The illustrative reviews do not demonstrate that this is the dominant source of disagreement, however, or exclude genuine differences between the included studies. Explicit routing and adaptation records make this contributor more visible and testable.

Compounding this is a conflation of two distinct constructs. Reporting completeness measures what an author wrote. Risk of bias measures what the study design permits us to conclude. A well-conducted study can be poorly reported, and a comprehensively reported study can be badly designed. CLAIM measures the first; QUADAS-3 and PROBAST+AI measure the second. Combining them into a single "quality" verdict, or ranking studies by CLAIM adherence, discards the distinction that makes either useful.

Scale of retrieval and the need for comparable appraisal

One indication of retrieval volume is available from published reviews. A 2025 review of caries detection on intraoral images identified 273 records and included 23 studies [15]; a review of plaque and gingivitis on photographs identified 3,307 records and included 23 [16]; a 2026 review of Faster R-CNN and Mask R-CNN reported screening 354 unique Faster R-CNN records [17]. These are focused reviews, but their search sources, strategies, and dates differ. The counts illustrate retrieval volume and should not be compared as estimates of a common eligible corpus.

The broad PubMed strategy reported in the earlier manuscript was rerun on 30 September 2026 through the NCBI Entrez interface, using artificial intelligence, dental, and imaging concept blocks without study-design filters and retaining the publication window of 1 January 2020 to 22 August 2026. It returned 5,357 records. This is the retrieval volume of one database on one execution date, not an eligible-study count, a precision estimate, or a measure of the resources required for appraisal. Appendix 1 provides the exact query, publication-date limits, execution date, count, and export verification details. The frozen record identifiers and metadata are retained in the authors’ revision archive for editorial verification; uploading them as editor-review files does not make them a published supplement. A systematic review would require its own prespecified, justified search and transparent reporting [38,39].

Interpreted narrowly, however, it illustrates the point of this review. Repeated reviews of a large literature may produce difficult-to-compare results when instrument selection and adaptations are not reported. Comparability requires that instrument selection, adaptation, and routing be specified in advance and reported in full, so that a later review can be checked against an earlier one rather than merely contradicting it.

A task-routed appraisal framework

There is a straightforward alternative. Route each eligible evaluation by the review question, study design, and task, apply an instrument within its stated scope, and report the results by route without pooling across them (Figure 1, Table 1).

**Figure 1:**
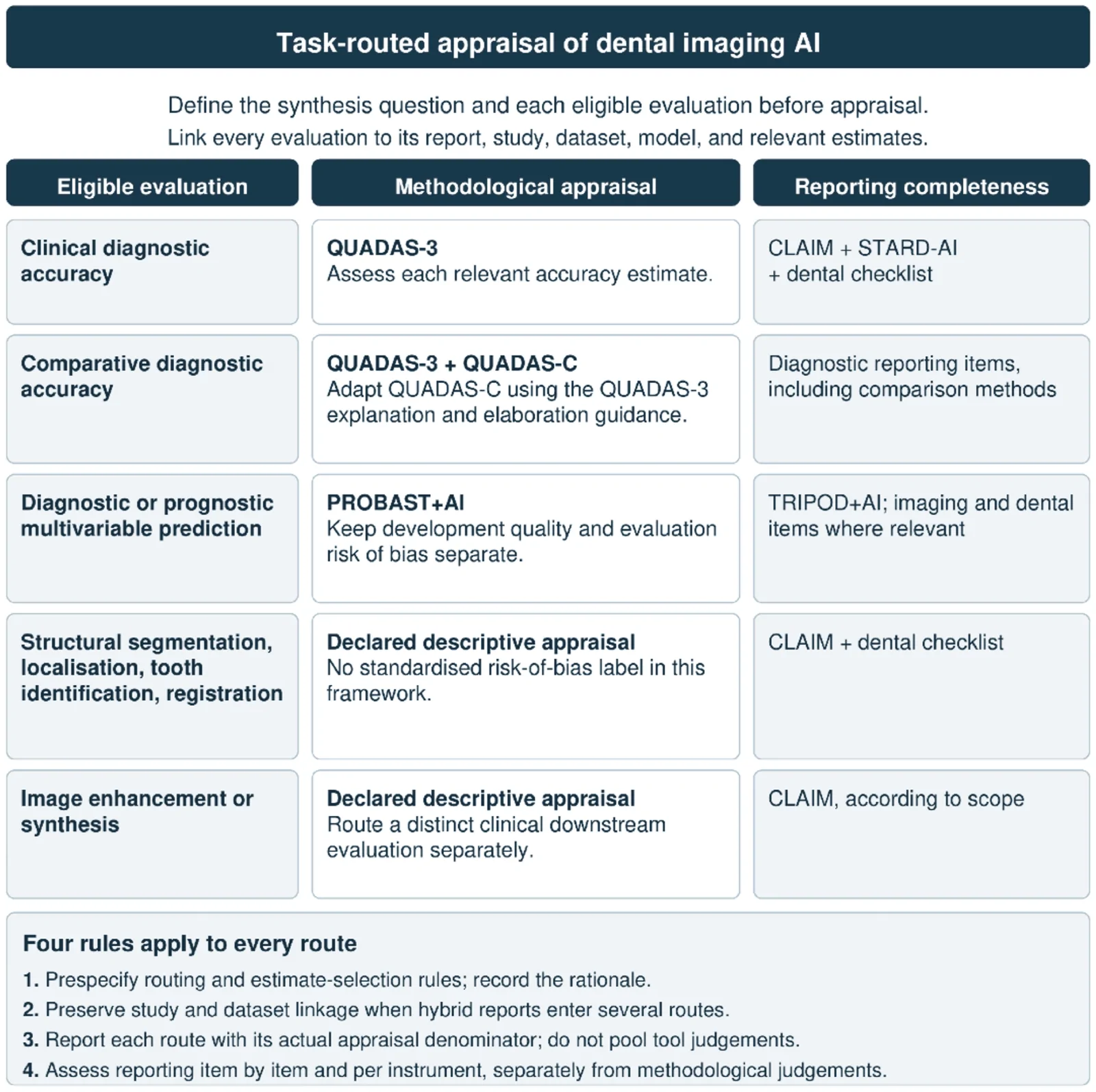
Task-routed appraisal of dental imaging artificial intelligence studies Each eligible evaluation is linked to its report, underlying study, dataset, and relevant estimates. Instrument selection depends on the synthesis question, study design, and endpoint. Hybrid reports may enter more than one route. Comparative appraisal uses QUADAS-C with adaptation for QUADAS-3 according to its explanation and elaboration guidance [13,23]. Reporting completeness and methodological judgements are presented separately QUADAS: Quality Assessment of Diagnostic Accuracy Studies; CLAIM: Checklist for Artificial Intelligence in Medical Imaging; STARD: Standards for Reporting of Diagnostic Accuracy Studies; PROBAST: Prediction Model Risk of Bias Assessment Tool; TRIPOD: Transparent Reporting of a Multivariable Prediction Model for Individual Prognosis or Diagnosis Original figure concept: Roksolana Mikhlin

**Table 1:** Task-routed appraisal framework for dental imaging artificial intelligence studies APPRAISE-AI: Appraisal of Artificial Intelligence Studies for Clinical Decision Support; CLAIM: Checklist for Artificial Intelligence in Medical Imaging; PROBAST+AI: Prediction Model Risk of Bias Assessment Tool, Artificial Intelligence Extension; QUADAS-C: Quality Assessment of Diagnostic Accuracy Studies, Comparative Extension; QUADAS-3: Quality Assessment of Diagnostic Accuracy Studies, Version 3; STARD-AI: Standards for Reporting of Diagnostic Accuracy Studies, Artificial Intelligence Extension; TRIPOD+AI: Transparent Reporting of a Multivariable Prediction Model for Individual Prognosis or Diagnosis, Artificial Intelligence Extension Table credits: Alina Shmal. The dental checklist is the 31-item checklist for artificial intelligence in dental research [19]

Eligible evaluation	Risk-of-bias/methodological route	Reporting-completeness instrument	Principal caveat
Accuracy of a specified diagnostic index test against a clinical reference standard	QUADAS-3, per relevant accuracy estimate [12,13]	CLAIM [8,9], STARD-AI [11], and the dental checklist [19], according to scope	Establish relevance to the synthesis question first. If one estimate is needed for a descriptive summary, use a prespecified primary-endpoint and test-set rule. Assess all estimates needed for substantive synthesis questions
Comparative accuracy: model versus clinician, model versus model, or assisted versus unassisted reading	QUADAS-3 for relevant estimates, plus QUADAS-C adapted using the QUADAS-3 guidance [13,23]	Same diagnostic reporting instruments, with comparison methods reported explicitly	Record the base tool and adaptations. QUADAS-C assesses comparison-specific bias, not applicability or indirect comparisons between separate single-test studies
Development or evaluation of a diagnostic or prognostic multivariable prediction model	PROBAST+AI, relevant development and evaluation components [10]	TRIPOD+AI [21]; CLAIM [9] and the dental checklist [19] where relevant	Development quality, evaluation risk of bias, and applicability are distinct judgements. A diagnostic target alone does not decide between this route and test-accuracy appraisal
Structural segmentation, localization, tooth identification, or registration with technical endpoints	Prespecified descriptive appraisal; APPRAISE-AI only where its intended scope is justified [27]	CLAIM [9] and the dental checklist [19]	This framework assigns no standardized risk-of-bias label. Report any item adaptation; an adapted APPRAISE-AI score is not the validated original
Image enhancement or synthesis without a clinical reference standard	Descriptive appraisal of acquisition, processing, and downstream evaluation [37]	CLAIM [9], according to scope	Technical image quality does not establish clinical usefulness. Appraise a separate clinical downstream evaluation under the corresponding route

Four rules govern the framework.

First, the routed unit is each eligible evaluation, defined by its task, target condition or structure, and test setting within a report. A hybrid report may contribute, for example, a segmentation evaluation and a clinical diagnostic evaluation, each appraised under the relevant route. For route-set summaries, a comparative evaluation enters the comparative route. Its component accuracy estimates still require QUADAS-3, but a separate single-test route is added only when a distinct eligible single-test evaluation exists. Where no primary task is stated, the reviewer routes the evaluations relevant to the synthesis question and records that omission. All assessments retain linked report, study, dataset, model, and estimate identifiers. Each route reports the numbers of unique studies, reports, evaluations, and appraised estimates or comparisons as appropriate. Proportions use the denominator of the actual appraisal unit: accuracy estimates for QUADAS-3, comparisons for QUADAS-C, and model evaluations for the evaluation component of PROBAST+AI. A study contributing several assessments is counted once in a study-level total; datasets shared between studies are flagged rather than treated as independent replications.

Second, routing and estimate-selection rules are specified in the protocol before appraisal. The decision for each evaluation and its rationale are recorded. Third, no overall risk of bias is pooled across routes. A single summary proportion of studies at high risk of bias is uninterpretable if it combines QUADAS-3 judgements, PROBAST+AI judgements, and descriptive features, because the three do not measure the same thing. Results are reported by route. Fourth, reporting completeness is assessed on every report regardless of route, at report level, and expressed as the proportion of applicable items reported. Three constraints follow, and they are the weakest part of any adherence measure.

Adherence proportions are descriptive summaries of what a paper states. They are not validated quality scores; they have no established thresholds, and studies should not be ranked by them. The instruments themselves say as much: CLAIM's authors note that it was not designed as a scoring tool [9]. What the numbers support is item-level reporting, which is where their diagnostic value lies: a corpus in which one item is reported by a tenth of studies tells an editor something a mean adherence figure does not.

Where several reporting instruments apply to one route, proportions are reported per instrument and never combined. The diagnostic route stacks CLAIM, STARD-AI and the dental checklist, and these overlap heavily on data provenance, partitioning and the reference standard, so a combined figure counts the same reported item three times and penalizes the same omission three times. Where two instruments disagree about whether an item applies to a given study, the reviewer records both judgements and states which instrument's scope was followed, rather than resolving the disagreement silently.

Item applicability determines the denominator of a reporting proportion; this is separate from clinical applicability in a risk-of-bias tool, and marking an item not applicable raises the reported proportion without anything changing in the paper. Not-applicable judgements should therefore be made independently by two raters, reconciled, and reported as counts alongside the proportions. Where a protocol adds a partial category to CLAIM's official Yes, No and Not Applicable response set, the item-level decision rules must be specified in advance, and both a strict analysis in which partial counts as not reported and a lenient sensitivity analysis must be presented.

Units of analysis: record, report, study, dataset, model, estimate

Task routing is only coherent if the unit being appraised is defined. Dental imaging artificial intelligence research routinely produces several publications from one dataset, several models within one publication, and several performance estimates per model. Bibliographic, study, and analytical units should be distinguished and preserved, including the evaluation used for routing.

A record is one database citation before de-duplication. A report is one publication. A study is one underlying investigation; reuse of a dataset does not automatically make two investigations the same study, and several reports may describe one study. A dataset is one image collection, identified by name, version, and provenance. A model is one trained algorithm or algorithm family evaluated in a report. An estimate is one combination of target condition, model version, test set, and decision threshold.

The dataset is the unit most often overlooked. Public dental image collections are now reused across many publications, and a review that treats a dozen reports built on the same public panoramic collection as a dozen independent studies overstates the evidence considerably. The overstatement is invisible in any count of studies. This is the non-independence problem that the record and study distinction handles, one level up, and it is separate from clustering within a dataset. Extraction should therefore record dataset name, version, and provenance, and whether the collection is public or institutional, and overlap in datasets across included studies should be flagged in the synthesis.

The intended instrument-to-unit mapping is as follows. CLAIM and the dental checklist are assessed at report level. QUADAS-3 is assessed for each relevant accuracy estimate, and QUADAS-C adds an assessment of each relevant test comparison. PROBAST+AI is assessed for the model, with development and evaluation parts distinguished. Companion reports, co-publications, corrections, letters, and preprints are linked rather than deleted, and the reason for inclusion, exclusion, or linkage is recorded.

This is not a bureaucratic refinement. Identical titles in a bibliographic export can denote a preprint and its journal version, a simultaneous co-publication in two journals, an ahead-of-print and issue record, or a Letter to the Editor carrying the same title as the primary article it comments on. For example, the tooth-numbering study by Estai and colleagues and the Letter to the Editor by Gurses and colleagues have the same title but distinct publication types and DOIs [40,41]. Treating any of these as a routine duplicate and deleting one silently corrupts both the count and the appraisal. Publication type determines eligibility; title similarity determines only that a relationship needs to be established.

What appraisal must capture that is specific to dentistry

General medical imaging instruments name the right domains but not the dental particulars that populate them. Four areas deserve explicit attention.

The Reference Standard is Usually Annotation, and Its Reliability is Usually Unquantified

 Reference standards depend on the target condition. In the dental reviews discussed here, many evaluations use annotations made by clinicians on the same images seen by the model [14,16,17]. This has a specific consequence. Evaluation against expert annotation measures agreement with those annotations; it does not independently establish accuracy against the underlying disease state, and a model that agrees closely with its annotators may be no closer to the disease than they were. It does not follow that a model can never outperform an individual annotator: assessed against a stronger, independent reference standard such as histology or clinical verification, a model may do so, and studies that make that comparison are appraising something different from studies that do not. Appraisal should therefore record the number of annotators, their stated experience, the calibration and consensus procedure, and whether an intra- or inter-annotator agreement statistic is reported.

Where a second modality verifies, as when caries scored on a panoramic radiograph is confirmed on bitewings, that verification is a design feature deserving credit, though it is not automatically a definitive reference standard; whether it is depends on the target condition and on how the verification was performed and interpreted. The variation on these points is wide and is invisible in a headline accuracy figure. In the most recent modality-stratified dental review, which included 17 studies, the reference standard domain was rated unclear in 8 of 17 (47.1%), largely because papers did not report whether the reference assessment was blinded to algorithm output or how rigorously the reference diagnosis was established; several included studies used a single annotator with no consensus procedure and no stated caries definition [17]. The review reports 47.1% in its Results section and Figure 5, with a denominator of 17 studies; the corresponding integer count is eight. Appendix 1 shows the derivation.

The analysis unit and the level of partition disjointness

 Dental studies report at tooth level, surface level, lesion level, image level, or patient level, and these are not interchangeable. When training and test sets contain images from the same patients, performance may be optimistic because information about those patients can cross the partition boundary. The critical reporting item is not simply that data were split, but at what level the split was disjoint. Where a study states only that images were divided in a given ratio, patient-level separation cannot be assumed.

Two distinct problems hide behind that single item and should be extracted separately. Patient overlap between training and test sets is a leakage problem: the model has seen the test patient, and the resulting estimate may be optimistic. Multiple teeth, surfaces or images from the same patient in the test set is a clustering problem: the observations are not independent, so the point estimate may be unbiased while its confidence interval can be too narrow if within-patient dependence is ignored. Patient-level separation of partitions solves the first and does nothing about the second. An extraction form should therefore capture both the level at which partitions were disjoint and whether the analysis accounted for within-patient dependence when estimating uncertainty; in practice the latter is reported far less often than the former.

The same precision is required of the word external. A test set drawn from the same institution at a later date, one drawn from a different centre, and one assembled independently of the developers probe different aspects of transportability: temporal stability, transfer across sites, and independence from the development team respectively [42]. They do not form a hierarchy in which the last guarantees generalizability; an independently assembled set from an unrepresentative population may support a weaker claim than a well-chosen multi-centre set. What the extraction form should record is which aspect was tested, not a rank.

Spectrum is shaped by exclusions that are rarely justified clinically

 Studies commonly exclude images with restorations, crowns, implants, overlapping contacts, motion artefact, or poor exposure, and some require the target condition to be present in every included case. Such exclusions can narrow the clinical spectrum and change the population to which the estimate applies. The parallel risk is site-specific confounding, in which a model learns features of the acquiring institution rather than of the disease. This was demonstrated in chest radiography and can be difficult to detect when evaluation is confined to one clinic [43]. Where eligibility required the presence of the target condition, or excluded difficult cases without a clinical rationale, the participant selection domain deserves close scrutiny, but the judgement is not automatic, and the two concerns should be separated. A narrowed spectrum limits applicability, which is a statement about the match between the study and a reader's clinical question. Whether it also biases the accuracy estimate depends on the mechanism: case-control style selection of unambiguous positives and healthy negatives can inflate accuracy, whereas excluding images that a clinician would also decline to read may be defensible if the intended use is stated accordingly.

Exclusions should be judged against the stated intended population and use, and against the instrument's own signalling questions, rather than triggering a high rating by category. In the same review, participant selection raised the greatest applicability concern of any domain, with 13 of 17 studies (76.5%) rated high or unclear [17]. The same Results section describes applicability concern as generally low, reports low reference-standard applicability concern in nine of 17 studies, and shows a more favorable index-test profile. These domain-level findings qualify its general summary. Flow and timing are risk-of-bias domains, not an applicability domain, in the assessment shown. Great and unclear concern also represent different judgements; a combined proportion should not be interpreted as evidence that every study was demonstrably inapplicable.

Acquisition characteristics differ by modality

 The evidence is also unevenly distributed. An umbrella review of caries diagnosis found that bitewing and periapical radiographs and near-infrared transillumination were covered by several of its included reviews, while optical coherence tomography and cone-beam computed tomography each appeared in two of the seven included reviews [44]. Where a modality has been studied more closely, acquisition has proved to matter: a review of caries detection on oral photographs found model performance varying with the capture device and with lesion severity [45]. The items that matter therefore differ enough between modalities that a generic extraction form will miss them (Table 2).

**Table 2:** Modality-specific appraisal considerations in dental imaging artificial intelligence Citations identify the relevant modality literature. Acquisition and risk items are proposed extraction considerations; the intraoral-scan and CBCT-specific checks are methodological recommendations rather than demonstrated effects from the cited radiograph study CBCT: cone-beam computed tomography; OCT: optical coherence tomography Table credits: Tamara Danko

Modality	Reference standard typically used	Acquisition items to extract	Principal spectrum and generalizability risk
Bitewing and periapical radiographs [14,44]	Expert annotation, sometimes with histological or cavitation verification [14]	Sensor type, exposure parameters, positioning device	Overlap and angulation exclusions; single-clinic sensor uniformity [14]
Panoramic radiographs [17,40]	Expert annotation, occasionally verified on bitewings or clinically [17]	Unit manufacturer, programme, patient positioning	Superimposition and ghost artefact; exclusion of restored dentitions [17]
Cone-beam computed tomography [2,44]	Specify the volumetric annotation or other reference used for the target condition	Voxel size, field of view, reconstruction and preprocessing pipeline	Proposed checks: metal artefact, scanner diversity, and patient/volume-level versus slice-level separation
Intraoral surface scans	Specify the mesh annotation or other geometric reference	Scanner model, mesh resolution, capture protocol	Proposed checks: decimation, preprocessing, and device-specific surface characteristics
Intraoral and extraoral photographs [16,45]	Clinical examination or expert annotation [16]	Camera, illumination, retraction, magnification	Illumination and color variability; tooth-surface unit definition [45]
Optical coherence tomography and near-infrared transillumination [44]	Histology in vitro; clinical or radiographic in vivo [14]	Device, wavelength, acquisition geometry	Small, often ex vivo samples; lesion verification method; limited external data [44]

Table 2 has a practical consequence: an extraction form for dental appraisal must be modality-aware. A single generic form will record the same fields for a cone-beam volume and a smartphone photograph, and will therefore miss the features that determine whether either result transfers.

Implications for authors, reviewers, and editors

Editors and reviewers of dental artificial intelligence manuscripts have identified limited external testing and overstated claims among the reported reasons for rejection, and have recommended routine use of reporting checklists [46]. A scoping review of ethics in dental artificial intelligence found approval, consent and data governance to be sparsely reported [47], and these items belong among the basics rather than in a closing paragraph. The framework above suggests where enforcement would be most efficient, and Table 3 sets out the items we would propose as prerequisites for peer review. They are recommendations, not established requirements, and each asks an author to disclose whether something was done rather than to have done it. It also marks the boundary of what retrospective accuracy studies can establish: moving a model from a reported metric to demonstrated clinical usefulness requires early-stage evaluation with clinicians in the loop, for which a separate reporting standard already exists [48].

**Table 3:** Minimum reporting set for dental imaging artificial intelligence submissions These 12 items are proposed editorial disclosure recommendations, not established journal requirements; justified non-applicability should be stated. Citations identify supporting guidance or evidence CI: confidence interval Table credits: Volodymyr Kachmar

Item	What must be stated	Why it is decisive
Number of contributing centres and devices	Explicit count, not "a university hospital"	Single-centre testing alone provides limited evidence of transfer across sites [7]
Partition method and level of disjointness	Patient, image, or tooth level, stated explicitly	Identifies potential patient or dataset leakage
Validation and test sets enumerated separately	Counts for each, and what each was used for	Test-set reuse for model selection risks optimistic performance estimates
Annotator number, experience, consensus procedure, and agreement statistic	Whether each was performed, and the value where it was	Clarifies the reference labels and their reliability; justified non-applicability is acceptable
External test set and its type	Absent, temporal, geographic, independently assembled, or a combination [42]	The test-set description states which aspect of transportability was examined
Eligibility exclusions with clinical rationale	Which images were excluded and why	Determines spectrum and applicability
Uncertainty around the primary metric	Confidence intervals, not point estimates alone, and whether within-patient clustering was accounted for	Shows precision and whether within-patient dependence was considered
Analysis unit and its clinical meaning	Tooth, surface, lesion, image, or patient	Determines what the number means to a clinician
Intended use and deployment stage	Standalone, assistive, or research only	Separates accuracy claims from clinical claims [48]
Code and data availability	Statement with a location or an explicit reason for restriction	Enables verification
Dataset identity and provenance	Name, version, and whether public or institutional	Reuse of public collections across studies creates non-independence that study counts hide
Ethics approval or waiver, consent where applicable, and data governance	Approval or waiver body and identifier; consent status or grounds for waiver; de-identification and authorised data use	Makes the governance basis explicit; retrospective use may require approval or a justified waiver [47]

For Authors

Select the reporting guideline by study type before writing and supply a completed checklist rather than merely citing it. State the intended use, clinical target, reference standard, partitioning, and limitations. Table 3 asks for disclosure of whether procedures were performed; it does not require every design to include the same procedures.

For Peer Reviewers

Establish the synthesis or clinical question, eligible evaluation, and analysis unit before selecting an appraisal instrument. Check that the reporting checklist and methodological tool address the actual design. Keep unreported information, an identified methodological flaw, and clinical inapplicability distinct.

For Editors

A completed reporting checklist can support submission checks. The 12 proposed disclosure items in Table 3 may help identify missing information before peer review, while allowing a stated rationale for non-applicability. Prospective clinical usefulness requires evidence beyond retrospective technical performance [48].

For Systematic Reviewers

Register the protocol, use the relevant PRISMA guidance [30,49], and specify routing and estimate-selection rules before appraisal. Report agreement from independent pre-consensus ratings, with paired counts and category marginals alongside any coefficient; post-consensus values do not estimate inter-rater agreement. Where an artificial intelligence system contributes to screening or appraisal, describe its role, archive its version, prompts, inputs, and outputs, and distinguish human-AI agreement from human-human agreement. Named human accountability for decisions and transparent reporting are consistent with the joint position of Cochrane, the Campbell Collaboration, JBI, and the Collaboration for Environmental Evidence [50].

Limitations

This review proposes a framework rather than establishing its routing reliability. The four authors independently reviewed and confirmed the AI-assisted editorial assessments during revision. Separately recorded item-level human decisions made before discussion are not reported, so human agreement coefficients, paired counts, and category marginals are not presented. The worked examples remain hypothetical. Testing on a defined sample, with independent pre-consensus decisions and transparent handling of hybrid studies, is needed before reproducibility can be claimed. Broader evaluation by reviewers who did not develop the framework would help assess its transportability.

The descriptive routes do not provide a standardised risk-of-bias judgement or a validated mapping to certainty-of-evidence domains. The framework has also not been evaluated for general-purpose multimodal models, especially where pretraining data or hosted-model changes are inaccessible. No corpus-level estimates of reporting completeness or risk of bias are reported. Prior reviews are used as published illustrations rather than independently re-appraised datasets, so instrument choice cannot be isolated from eligibility, population, and reviewer differences. The exact counts derived from rounded percentages in one review use its stated denominator of 17 and do not constitute a new appraisal. The broad historical PubMed retrieval is unscreened and illustrates retrieval volume only. Instrument availability will continue to change; the lookup dates and the versions used should be retained. Finally, the modality-specific extraction considerations are recommendations, not quantitatively validated predictors of appraisal conclusions.

## Conclusions

Dental imaging artificial intelligence research includes clinical test evaluations, prediction models, and technical imaging tasks that require different appraisal approaches. Updated instruments provide defined scopes, but their selection and adaptation need to be explained when these designs appear in the same review. Published differences in risk-of-bias judgements may reflect instrument choices as well as differences in eligible studies and reviewer interpretation; this review does not identify their relative contributions. The proposed framework links appraisal to each eligible evaluation and relevant synthesis question, keeps reporting completeness separate from methodological judgements, and records the dental particulars needed to interpret performance: annotation provenance, analysis unit, partition separation, clustering, dataset overlap, exclusions, and acquisition characteristics. Hybrid evaluations are linked to their underlying studies, and results are reported separately by route with explicit denominators. This approach is intended to improve transparency and comparability. Its routing reliability and its contribution to reproducible appraisal remain to be tested.

## Appendices

Supplementary file 1. Search documentation and revision verification

Manuscript: Appraising Artificial Intelligence Studies in Dental and Maxillofacial Imaging: A Task-Routed Framework for Reporting Completeness and Risk of Bias

This is a narrative methodological review. The queries below document identification and verification of guidance and illustrative reviews; they do not define an exhaustive eligible corpus of primary studies. No systematic primary-study screening, new risk-of-bias appraisal, or meta-analysis was performed. Historical search information is distinguished from the dated repeat queries completed for this revision.

Part 1. Appraisal instruments and reporting guidance

The initial search description supplied with the manuscript recorded an update on 24 September 2026. It identified instruments through the EQUATOR Network reporting-guidelines library, reference lists, and targeted PubMed lookups. Revision checks were completed on 30 September 2026 using Crossref bibliographic records, PubMed, the relevant published statements, and official instrument resources where available. The following DOI queries were executed in PubMed through NCBI Entrez ESearch, without language or date filters. These were known-record verification queries, not an exhaustive search for every relevant instrument (Table 4).

**Table 4:** Known-record PubMed DOI verification of appraisal instruments and reporting guidance Each row reports the records returned by the stated DOI query in PubMed through NCBI Entrez ESearch on 30 September 2026, without date or language limits. These checks verify known records, not an exhaustive guidance corpus. The dental AI checklist returned no record in this execution; it was verified through Crossref and EQUATOR AI: artificial intelligence; DOI: digital object identifier; NCBI: National Center for Biotechnology Information; EQUATOR: Enhancing the Quality and Transparency of Health Research; CLAIM: Checklist for Artificial Intelligence in Medical Imaging; STARD: Standards for Reporting of Diagnostic Accuracy Studies; QUADAS: Quality Assessment of Diagnostic Accuracy Studies; QUADAS-C: Quality Assessment of Diagnostic Accuracy Studies, Comparative Extension; PROBAST: Prediction Model Risk of Bias Assessment Tool; TRIPOD: Transparent Reporting of a Multivariable Prediction Model for Individual Prognosis or Diagnosis; LLM: large language model; APPRAISE-AI: Appraisal of Artificial Intelligence Studies for Clinical Decision Support; PRISMA: Preferred Reporting Items for Systematic Reviews and Meta-Analyses; PRISMA-S: PRISMA extension for reporting literature searches in systematic reviews; PRESS: Peer Review of Electronic Search Strategies; DECIDE-AI: Developmental and Exploratory Clinical Investigations of Decision Support Systems Driven by Artificial Intelligence

Instrument/source	Exact PubMed verification query	Records retrieved
CLAIM original	"10.1148/ryai.2020200029"[doi]	1
CLAIM 2024	"10.1148/ryai.240300"[doi]	1
STARD 2015	"10.1136/bmj.h5527"[doi]	1
STARD-AI	"10.1038/s41591-025-03953-8"[doi]	1
QUADAS-2	"10.7326/0003-4819-155-8-201110180-00009"[doi]	1
QUADAS-C	"10.7326/M21-2234"[doi]	1
QUADAS-3	"10.7326/ANNALS-25-02104"[doi]	1
QUADAS-3 explanation	"10.7326/ANNALS-25-04943"[doi]	1
PROBAST original	"10.7326/M18-1376"[doi]	1
PROBAST+AI	"10.1136/bmj-2024-082505"[doi]	1
TRIPOD+AI	"10.1136/bmj-2023-078378"[doi]	1
TRIPOD-LLM	"10.1038/s41591-024-03425-5"[doi]	1
APPRAISE-AI	"10.1001/jamanetworkopen.2023.35377"[doi]	1
Radiomics Quality Score source	"10.1038/nrclinonc.2017.141"[doi]	1
Dental AI checklist	"10.1016/j.jdent.2021.103610"[doi]	0
PRISMA 2020	"10.1136/bmj.n71"[doi]	1
PRISMA-S	"10.1186/s13643-020-01542-z"[doi]	1
PRESS	"10.1016/j.jclinepi.2016.01.021"[doi]	1
DECIDE-AI	"10.1038/s41591-022-01772-9"[doi]	1

The dental checklist's DOI query returned no record in this execution; its bibliographic record and listing were verified through Crossref and EQUATOR. A zero result from this known-record PubMed query is not evidence that the instrument was unpublished. The EQUATOR entry lists PMID 33631303. The direct record request did not return an article in the revision check, so no PubMed content verification is claimed for that record.

The EQUATOR listing checked was: https://www.equator-network.org/reporting-guidelines/artificial-intelligence-in-dental-research-checklist-for-authors-reviewers-readers/

The additional guidance requested by the reviewer was checked using the combined PubMed query below, without language or date limits. It retrieved four records, whose titles, abstracts, and publication details were examined.

"10.1001/jama.2017.19163"[doi] OR "10.1038/s41591-020-1034-x"[doi] OR "10.1038/s41591-020-1037-7"[doi] OR "10.1136/bmj.39500.677199.AE"[doi]

QUADAS-AI Status Check

The query "QUADAS-AI"[Title/Abstract], executed on 30 September 2026 without date or language limits, retrieved 60 records. The returned bibliographic records were inspected to distinguish development papers from reviews reporting that they had used QUADAS-AI. The 2021 announcement and 2024 development protocol were identified; no final tool statement was identified in this lookup. This is a dated search finding, not a claim that every possible publication source was exhaustively checked.

QUADAS-C Compatibility Check

The University of Bristol QUADAS-C page states that the tool was originally developed alongside QUADAS-2 and now recommends use alongside QUADAS-3,3 with adaptation described in the QUADAS-3 explanation and elaboration document. It also states that QUADAS-C assesses comparative risk of bias, not applicability or indirect between-study comparisons. The page was checked on 30 September 2026: https://www.bristol.ac.uk/population-health-sciences/projects/quadas/quadas-c/

Part 2. Query for illustrative dental reviews

Database and platform: PubMed through NCBI Entrez ESearch.

Revision execution date: 30 September 2026. The initial search description recorded 24 September 2026; the executable repeat query is provided below.

Limits: publication date from 1 January 2020; no language limit; publication types: Systematic Review or Meta-Analysis. The open upper publication-date bound is reproduced from the supplied strategy.

Records retrieved in the repeat execution: 103.

Complete query:

("artificial intelligence"[tiab] OR "machine learning"[tiab] OR "deep learning"[tiab] OR "neural network"[tiab] OR "neural networks"[tiab] OR convolutional[tiab]) AND (dental[tiab] OR dentistry[tiab] OR tooth[tiab] OR teeth[tiab] OR caries[tiab] OR periodont*[tiab] OR endodont*[tiab] OR orthodont*[tiab] OR maxillofacial[tiab] OR "dental implant"[tiab] OR "dental implants"[tiab]) AND (radiograph*[tiab] OR imaging[tiab] OR image*[tiab] OR photograph*[tiab] OR CBCT[tiab] OR "cone beam"[tiab] OR panoramic[tiab] OR bitewing[tiab] OR periapical[tiab] OR cephalometr*[tiab] OR "intraoral scan"[tiab] OR "optical coherence tomography"[tiab] OR transillumination[tiab]) AND ("risk of bias"[tiab] OR QUADAS[tiab] OR PROBAST[tiab] OR CLAIM[tiab] OR "quality assessment"[tiab] OR "quality appraisal"[tiab]) AND ("2020/01/01"[Date - Publication] : "3000"[Date - Publication]) AND ("Systematic Review"[Publication Type] OR "Meta-Analysis"[Publication Type])

The original description listed “Systematic Review; Meta-Analysis” as interface filters. The repeated query expresses their intended union explicitly with OR. Reviews were selected purposively to illustrate different tasks, modalities, and appraisal practices; there was no systematic eligibility screening or prespecified representative sampling. Additional methodological sources were identified by targeted lookups and citation checking. No claim is made that the 103 retrieved records constitute the whole supporting literature.

Verification of the Quoted Illustrative Findings (Table 5)

**Table 5:** Verification of illustrative findings reported in four dental artificial intelligence reviews Findings were checked against the source locations stated in the table and manuscript references 14–17. They reflect the original reviews’ eligibility criteria and appraisal procedures; no new risk-of-bias assessments were performed. Denominators vary between reviews and findings. The RQS result reported by Lam et al., 268/360 (74.4%), concerns 10 radiographic studies rather than all 17 included studies QUADAS-2: Quality Assessment of Diagnostic Accuracy Studies, Version 2; GRADE: Grading of Recommendations Assessment, Development and Evaluation; R-CNN, region-based convolutional neural network; RQS: Radiomics Quality Score

Review	Findings used in the manuscript	Source and interpretation
Mohammad-Rahimi et al., caries detection, 2022 [14]	11/42 studies low risk across all QUADAS-2 domains	Count and denominator reported in the published abstract; no new appraisal
Noor Uddin et al., intraoral-image caries, 2025 [15]	273 records identified; 23 studies included; 19 low risk and four high risk	Published abstract; “identified” is used rather than assuming all 273 were screened at one stage
Moharrami et al., plaque/gingivitis photographs, 2025 [16]	3,307 records identified; 23 studies included; 11 low risk and nine low applicability concern across all domains	Published abstract and full text; GRADE was used alongside QUADAS-2
Lam et al., Faster R-CNN/Mask R-CNN, 2026 [17]	17 studies included; 354 unique Faster R-CNN records screened; unclear reference-standard bias 47.1%; high/unclear participant-selection applicability concern 76.5%; RQS 74.4% (268/360)	Results sections 3.1–3.3 and Figure 5. RQS denominator concerns ten radiographic studies, not all 17

Exact Integer Counts Derived From the Source Percentages

Lam et al. state a denominator of 17 studies and report the percentages in the Results section. The manuscript presents the uniquely compatible integer counts, rather than claiming a new extraction of independent domain assessments (Table 6):

**Table 6:** Consistency check of integer counts derived from published percentages Both calculations use the source denominator of 17 included studies, reported in Results section 3.3 and Figure 5 of Lam et al. [17]. The counts are the integers compatible with the published percentages rounded to one decimal place. They are arithmetic derivations, not newly extracted independent domain assessments. “High or unclear” retains the source’s combined applicability category and does not equate these distinct judgements

Domain and judgement	Source percentage	Integer count	Check
Reference-standard risk of bias: unclear	47.10%	8 of 17	8/17 × 100 = 47.0588%, rounded to 47.1%
Participant-selection applicability: high or unclear	76.50%	13 of 17	13/17 × 100 = 76.4706%, rounded to 76.5%

The source also reports low reference-standard applicability concern in nine of 17 studies and a more favorable index-test applicability profile. Flow and timing are not an applicability domain in the displayed assessment. High and unclear applicability concerns are not interchangeable judgements.

The identical-title example uses the independently checked article by Estai et al. (DOI 10.1259/dmfr.20210296) and Letter to the Editor by Gurses et al. (DOI 10.1259/dmfr.20220128). It is a bibliographic example, not a result attributed to an undocumented pilot search or to a title-normalization analysis of the historical export.

Part 3. Broad PubMed retrieval used to describe scale

Purpose: to describe retrieval volume for a sensitive artificial-intelligence, dental, and imaging strategy. The result is not an eligible-study count, a precision estimate, or an appraisal-resource estimate.

Database and revision platform: PubMed, NCBI Entrez ESearch; citation metadata obtained through ESummary.

Revision execution date: 30 September 2026.

Fixed publication window: 1 January 2020 to 22 August 2026, inclusive, using the supplied PubMed publication-date expression.

Other limits: no language, publication-type, or study-design filter. No eligibility screening was performed.

Records retrieved and exported: 5,357 unique PMIDs. This is the figure used in the revised manuscript.

Complete query, repeated without changing its concept blocks or publication-date limit:

(("Artificial Intelligence"[Mesh] OR "Machine Learning"[Mesh] OR "Deep Learning"[Mesh] OR "artificial intelligence"[tiab] OR "machine learning"[tiab] OR "deep learning"[tiab] OR "neural network"[tiab] OR "neural networks"[tiab] OR convolutional[tiab] OR transformer*[tiab] OR "computer aided"[tiab] OR "computer-aided"[tiab]) AND (("Radiography, Dental"[Mesh] OR "Cone-Beam Computed Tomography"[Mesh] OR "Photography, Dental"[Mesh]) OR ((dental[tiab] OR dentistry[tiab] OR tooth[tiab] OR teeth[tiab] OR oral[tiab] OR maxillofacial[tiab] OR caries[tiab] OR periodont*[tiab] OR endodont*[tiab] OR orthodont*[tiab] OR "dental implant"[tiab] OR "dental implants"[tiab]) AND (radiograph*[tiab] OR image*[tiab] OR imaging[tiab] OR photograph*[tiab] OR CBCT[tiab] OR "cone beam"[tiab] OR panoramic[tiab] OR bitewing[tiab] OR periapical[tiab] OR cephalometr*[tiab] OR "intraoral scan"[tiab] OR "intraoral scans"[tiab] OR "optical coherence tomography"[tiab] OR "near infrared"[tiab] OR transillumination[tiab])))) AND ("2020/01/01"[Date - Publication] : "2026/08/22"[Date - Publication])

The earlier manuscript reported 5,292 records from a query executed on 22 August 2026. The repeat execution is reported separately and does not retrospectively certify that historical count. Publication-date limits do not freeze the contents of a database: records can be added or revised after the nominal publication period. The observed difference cannot be attributed to a particular indexing mechanism without the original historical export. No historical DOI/PMCID completeness statistics, duplicate-title groups, or unverified historical checksums are used as evidence in this revision.

Archive Supplied With this Revision

The complete metadata export is 07_Verified_PubMed_Export_2026-09-30.csv. The companion file 08_Verified_Search_Log_2026-09-30.json contains the literal query, returned PubMed query translation, complete PMID list, execution date, and export verification details.

CSV SHA-256: 275e9f12afc95ceffc9c54d52932374034e8ddcf0b08793ff0baeb0feec3eac1.

All 5,357 rows have distinct PMIDs. The archive establishes what this repeat execution retrieved; it does not establish eligibility.

Descriptive Publication-Year Fields in the Export

The year below is the first four characters of the ESummary PubDate field. It is an export descriptor, not an additional publication-date filter. PubMed date searching can use dates that differ from this display field; therefore, a displayed year outside 2020-2026, if present, is retained and explained rather than silently deleted (Table 7).

**Table 7:** Distribution of exported PubMed records by the displayed ESummary publication year Counts describe 5,357 unique PubMed identifiers exported from the broad query executed on 30 September 2026, with a fixed publication-date window of 1 January 2020 to 22 August 2026. Year is the first four characters of the PubDate display field returned by ESummary, the NCBI summary-record service; it is not an additional date filter. Records were not screened for eligibility, so these counts describe retrieval volume rather than included studies NCBI: National Center for Biotechnology Information; PMID: PubMed identifier; PubDate: displayed publication-date field

ESummary publication year	Records
2020	272
2021	380
2022	531
2023	630
2024	898
2025	1,327
2026	1,319
Total	5,357

Other databases and limits of inference

Scopus, Web of Science Core Collection, and Embase were not searched for this documented broad retrieval. No counts are reported or inferred for them. A formal evidence review needs its own justified sources, registered methods where appropriate, and a complete search report. The strategies here identify guidance, provide illustrative reviews, and establish the dated retrieval volume; they do not support corpus-level claims about study quality, eligibility, or the work needed for appraisal.
